# Bevacizumab as treatment option for recurrent respiratory papillomatosis: a systematic review

**DOI:** 10.1007/s00405-022-07388-6

**Published:** 2022-04-24

**Authors:** Louis Pogoda, Fuat Ziylan, Diederik P. J. Smeeing, Frederik G. Dikkers, Rico N. P. M. Rinkel

**Affiliations:** 1 Department of Otorhinolaryngology-Head and Neck Surgery, Amsterdam University Medical Center, Location VUmc, De Boelelaan 1117, 1081 HV Amsterdam, The Netherlands; 2 Department of Surgery, University Medical Center Utrecht, Heidelberglaan 100, 3584 CX Utrecht, The Netherlands; 3 Department of Otorhinolaryngology-Head and Neck Surgery, Amsterdam University Medical Center, Location AMC, Meibergdreef 9, 1105 AZ Amsterdam, The Netherlands

**Keywords:** Recurrent respiratory papillomatosis, Larynx papillomatosis, Human papilloma virus, Bevacizumab, Avastin®, Vascular endothelial growth factor

## Abstract

**Purpose:**

To this day, there is no cure for recurrent respiratory papillomatosis (RRP). Multiple surgical procedures are performed to achieve symptom relief and prevention of airway obstruction. A promising drug for RRP is the vascular endothelial growth factor (VEGF) binding antibody bevacizumab. This chemotherapeutic agent has an angiogenesis-inhibiting effect which inhibits tumor growth. The objective of this review was to investigate the efficacy of bevacizumab as treatment option for RRP, and to explore the difference of its effects between intralesional and systemic treatment.

**Methods:**

A systematic search was conducted in Cochrane, PubMed, and Embase. Articles were included if bevacizumab treatment was given intralesionally and/or systemically. The methodological quality of the studies was assessed using the CAse REport (CARE) guidelines.

**Results:**

Of 585 unique articles screened by title and abstract, 15 studies were included, yielding a total of 64 patients. In 95% of the patients treated with systemic bevacizumab, the post-bevacizumab surgical interval was considerably prolonged. More than half of them did not need any surgical intervention during mean follow-up of 21.6 months. Treatment with intralesional bevacizumab showed a lower efficacy: in 62% of the patients, the post-bevacizumab surgical interval (mean, 1.8 months follow-up) was extended when compared to the interval before the treatment.

**Conclusion:**

Systemically and intralesionally administered bevacizumab are effective treatment options for severe RRP. A systemic administration might be the treatment of first choice. Further prospective research with long term follow-up is advocated to elucidate this important topic.

## Introduction

Recurrent respiratory papillomatosis (RRP) is a rare disease of the respiratory mucosa and is characterized by the recurrent growth of papillomas at the sites of the epiglottis, supraglottis, plicae vocalis, subglottis, the tracheobronchial tract, and lung parenchyma. It is mainly caused by an infection with the human papilloma virus (HPV) type 6 or 11 [1, 2]. In 1–2% of the cases, tumor growth is caused by HPV type 16 or 18 and, therefore, considered to be premalignant [1, 2].

RRP can arise at any age but has a typical, trimodal age distribution with peaks around 7, 35 and 64 years [3]. Clinically, a distinction is made between the juvenile and adult type of onset, with an age of 18 years as the limit [3]. The prevalence of the juvenile-onset type (JoRRP) is estimated to be around 0.75—4 per 100,000. In addition, a juvenile manifestation is associated with a more aggressive course, multiple lesions and a higher risk of recurrence [4]. The prevalence of the adult-onset type (AoRRP) is estimated to be around 2 per 100,000 [1, 2].

Abnormal cell proliferation at the aforementioned predilection sites explains the typical symptom pattern with dysphonia, dyspnea, chronic coughing, stridor or screeching breathing. The severity and exact course of the disease differ per patient and are very unpredictable. Symptomatology can be mild and slowly progressive requiring little treatment. Contrary, the disease can also behave more aggressively, which requires rapid and repeated intervention [1, 2, 5].

To this day, there is no cure for RRP. The aim of all therapies is symptom relief and prevention of airway obstruction. Surgery using cold steel, CO_2_ laser or microdebrider are the most commonly used techniques, but multiple procedures are no exception given the recurrent nature of RRP [1, 2, 5]. As a result, the cumulative risk of general anesthesia and iatrogenic complications increases, the high surgery frequency leads to greater absenteeism at school or work and can subsequently lead to social and financial problems [1, 2, 6].

Over the past decade, new adjuvant therapies against RRP have been increasingly reported in literature [1, 2, 5, 7, 8]. A promising drug is the vascular endothelial growth factor (VEGF)-binding antibody bevacizumab. It is a chemotherapeutic agent with an angiogenesis-inhibiting effect that inhibits tumor growth [1, 2, 5]. Thus, it is hypothetically less likely to require repetitive surgery when given intralesionally or systemically. Today’s literature includes various case reports regarding bevacizumab treatment for RRP [9–23]. However, the majority of authors reported their experiences almost exclusively in selected patients with advanced to severe papillomatosis, and high quality studies and clinical trials that objectify the efficacy of bevacizumab for RRP are lacking [24]. The goal of this systematic review was to give an overview of the available literature concerning the efficacy of bevacizumab for RRP, and to differ between intralesional and systemic treatment.

## Materials and methods

The review was performed in accordance with the Preferred Reporting Items for Systematic Reviews and Meta-Analyses (PRISMA) statement [25, 26].

### Search strategy

In this systematic review, an electronic search was performed using the Cochrane, PubMed, and EMBASE electronic databases on 21 February 2022. Keywords used for the search included various synonyms and types for bevacizumab and RRP. The search strings can be found in Appendix 1.

### Selection criteria

Titles and abstracts were screened independently by two authors. After title and abstract screening, potentially valuable articles were read in full text. Articles were included if written in English, Dutch, German, Spanish, Portuguese, Turkish or Russian language. Case reports were included if bevacizumab was administered intralesional or systemic. Studies were excluded if it investigated concerned animal studies, opinion papers, poster presentations. Consensus on inclusion and exclusion was reached through discussion between the authors. If no consensus could be reached, a third author was consulted. References and citing articles were screened for additional studies.

### Quality assessment

The methodological quality of the studies was independently assessed by two authors using the CARE (CAse REport) guidelines, to assess the risk of bias in the included studies [27]. Consensus on quality assessment was reached after discussion between the authors.

### Data extraction

Study characteristics and outcome data of the included studies were extracted. Additionally, the following data were extracted: onset of disease, prior treatments, type of bevacizumab treatment, affected sites, surgical interval and number of procedures before and after bevacizumab treatment, Derkay score before and after bevacizumab treatment, treatment dose, initial and final dosing interval, treatment cycles, duration of follow-up, and complications.

## Results

### Search results and selection process

**Fig. 1:**
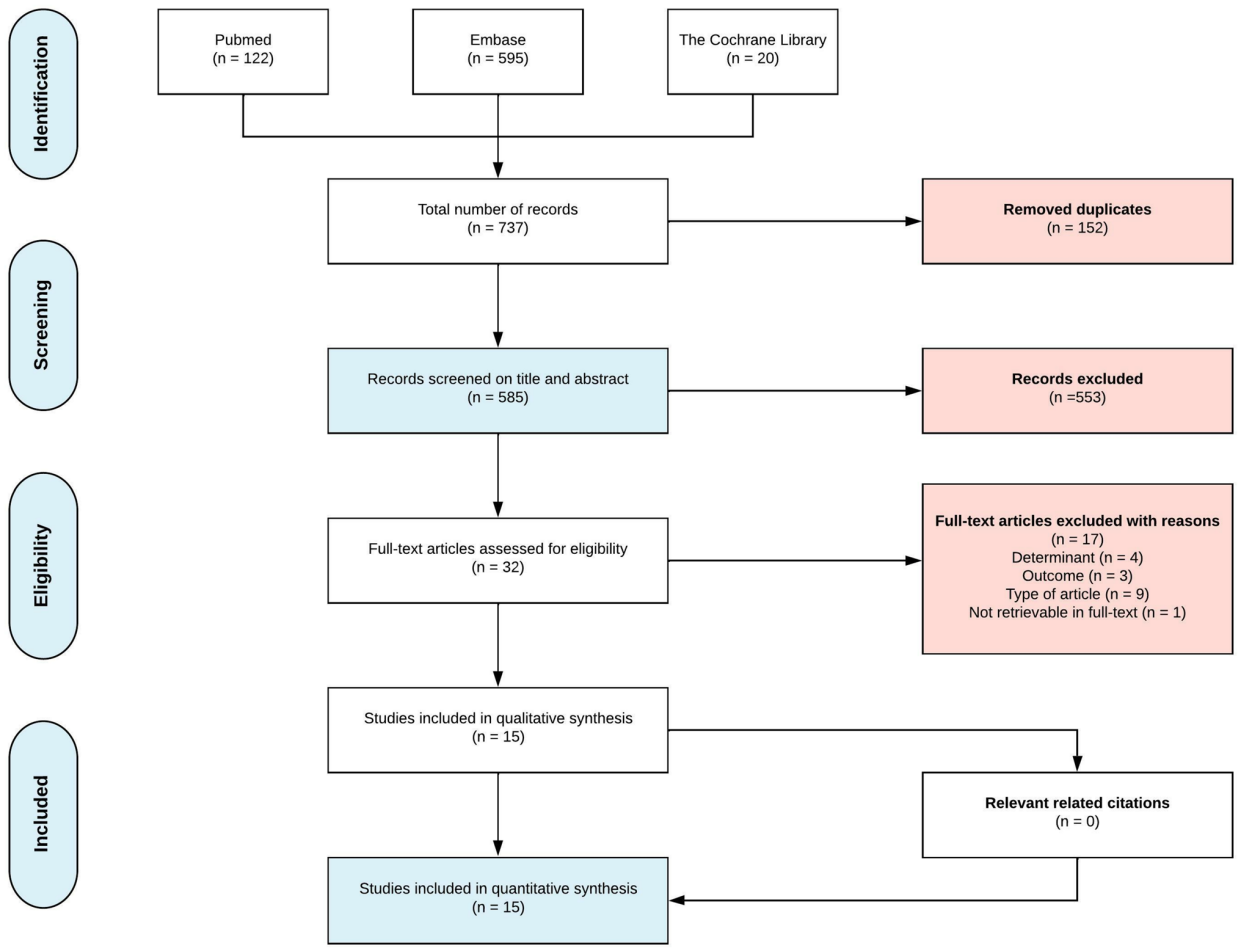
Flowchart of inclusion of relevant publications for use of systemic and intralesional bevacizumab in treatment of recurrent respiratory papillomatosis

A total of 585 articles were retrieved after removing duplicates. After title and abstract screening, 32 articles were assessed for eligibility in full text. No language restrictions were found. In total, fifteen studies were deemed eligible and critically appraised [9–23]. Reviewing of references and citation tracking did not result in additional relevant articles. A flowchart with the performed selection process is shown in Fig. 1.

### Quality assessment

Overall, twelve studies scored a moderate [10–13, 15, 16, 18–23], and three studies scored a high risk of bias [9, 14, 17]. The results of the critical appraisal are shown in Table 1.

**Table 1:** Critical appraisal of all manuscripts fulfilling inclusion criteria for use of systemic and intralesional bevacizumab in treatment of recurrent respiratory papillomatosis, according to the CARE guidelines [26]

Case report/series	Titel	Key-words	Abstract	Introduction	Patient information	Clinical findings	Timeline	Diagnostic assistant	Therapeutic intervention	Follow-up and outcomes	Discussion	Patient perspective	Informed consent	Risk of bias
Nagel et al. 2009	S	NS	S	PS	S	S	NS	S	S	PS	NS	NS	NS	H
Maturo et al. 2010	PS	NS	PS	S	S	S	NS	PS	S	PS	S	NS	S	M
Rogers et al. 2013	PS	NS	S	S	S	S	NS	PS	S	PS	S	NS	S	M
Mohr et al. 2014	PS	PS	S	S	S	S	NS	S	S	PS	NS	NS	S	M
Sidell et al. 2014	PS	PS	PS	S	S	S	NS	S	S	NS	PS	NS	S	M
Bedoya et al. 2017	PS	PS	S	S	S	S	NS	S	S	PS	PS	NS	S	M
Best et al. 2017	PS	PS	S	PS	S	S	NS	PS	S	S	S	NS	NS	M
Zur et al. 2017	PS	NS	S	PS	S	S	NS	S	S	S	PS	NS	S	M
Carnevale et al. 2019	PS	PS	PS	S	S	S	NS	S	S	S	PS	NS	S	M
Cuestas et al. 2019	PS	PS	PS	S	S	S	NS	S	S	S	S	NS	S	M
Baday et al. 2020	PS	PS	PS	S	S	S	NS	S	S	PS	PS	NS	NS	H
Gates et al. 2020	PS	PS	S	PS	S	S	S	S	S	S	PS	NS	NS	M
Hamdi et al. 2020	S	PS	PS	NS	S	S	NS	PS	S	PS	PS	NS	S	H
Tkaczuk et al. 2020	PS	PS	S	S	S	S	NS	S	S	S	PS	NS	S	M
Enrique et al. 2021	S	S	PS	S	S	S	NS	PS	S	PS	S	NS	NS	M

*S* satisfying, *PS* partially satisfying, *NS* not satisfying, *M* moderate, *H* high

### Baseline characteristics of included studies

The baseline characteristics of the included studies are shown in Table 2. The results of 64 patients were included. The majority of participants (54 out of 64) was diagnosed with JoRRP, and in ten patients RRP arose during adulthood. Bevacizumab treatment was provided systemically in 43 patients, whereas 21 patients received intralesional treatment. In the period before the initiation of bevacizumab, other treatments were performed. Surgical procedures were performed in all patients. Additionally, some participants underwent adjuvant treatment. This considered Gardasil® (*n* = 5), propranolol (*n* = 3), celecoxib (*n* = 5), leflunomide (*n* = 1), indole-3-carbinol (*n* = 3), interferon alpha (*n* = 15), and cidofovir (*n* = 26). In three studies, a total of 16 patients underwent intralesional bevacizumab injections prior to a periodical, systemic treatment [9, 11, 20].

**Table 2:** Chronologically listed baseline characteristics of all manuscripts fulfilling inclusion criteria for use of systemic and intralesional bevacizumab in treatment of recurrent respiratory papillomatosis

Case report/series	Total cases n, (JoRRP/ AoRRP)	Patients with prior treatment (*n*)									Examined bevacizumab treatment
		Gardasil	Propranolol	Celecoxib	Lefluno mide	Indole-3 Carbinol	Interferon alpha	Cidofovir	Surgery	Intralesional bevacizumab	
Nagel et al. 2009	1, (1/0)	0	0	0	0	0	1	1	1	0	Systemic
Maturo et al. 2010	3, (3/0)	0	0	0	0	0	0	3	3	0	Intralesional
Rogers et al. 2013	10, (10/0)	0	0	0	0	0	0	4	10	0	Intralesional
Mohr et al. 2014	5, (2/3)	0	0	1	0	0	1	1	5	0	Systemic
Sidell et al. 2014	8, (8/0)	0	0	0	0	0	0	0	8	0	Intralesional
Bedoya et al. 2017	2, (0/2)	1	0	0	0	0	0	1	2	0	Systemic
Best et al. 2017	8, (6/2)	1	1	3	1	1	4	7	8	2	Systemic
Zur et al. 2017	1, (1/0)	1	1	1	0	0	1	1	1	0	Systemic
Carnevale et al. 2019	2, (2/0)	1	0	0	0	0	1	1	2	0	Systemic
Cuestas et al. 2019	1, (1/0)	0	0	0	0	0	0	0	1	0	Systemic
Baday et al. 2020	3, (3/0)	0	0	0	0	0	0	1	3	2	Systemic
Gates et al. 2020	1, (1/0)	0	0	0	0	0	0	0	1	0	Systemic
Hamdi et al. 2020	2, (2/0)	0	0	0	0	1	1	2	2	0	Systemic
Tkaczuk et al. 2020	14, (11/3)	1	1	0	0	1	6	3	14	12	Systemic
Enrique et al. 2021	3, (3/0)	0	0	0	0	0	0	1	3	0	Systemic
Total	64, (54/10)	5	3	5	1	3	15	26	64	16	-

*JoRRP* juvenile onset type of recurrent respiratory papillomatosis, *AoRRP* adult onset type of recurrent respiratory papillomatosis, *n* number

### Efficacy of systemic bevacizumab

The outcomes of the studies describing efficacy of systemic bevacizumab are presented in Table 3. In 95% of the cases (41 out of 43), the post-bevacizumab surgical interval was considerably prolonged. More than half of the patients (24 out of 43) did not require any surgical intervention for RRP during follow-up anymore. The surgical interval before initiation of bevacizumab ranged from 3 to 48 weeks [9, 11, 17, 20, 22, 23]. Some studies did not report this interval but the number of surgical procedures received, which ranged from 5 to 47 [10, 12, 13, 16]. One study reported the number of surgical interventions in the year prior to bevacizumab (range from 2 to 9) without further details [20]. In 40% of the patients (17 out of 43), surgical interventions were still required after bevacizumab treatment to achieve disease control. However, the post-bevacizumab surgical interval (range 3–17 months) was considerably longer compared to that before treatment (range 1–8.6 weeks). In one case malignant transformation of RRP occurred after three treatment cycles and led to discontinuation of bevacizumab [16]. No mention was made of HPV type in that case. Another case showed poor treatment effects of systemic bevacizumab [13]. Systemic and surgical treatments were given simultaneously during the entire follow-up period due to the severity of RRP.

**Table 3:** Treatment effects of twelve studies, listed per patient, reporting the use of systemic bevacizumab as adjunct therapy for recurrent respiratory papillomatosis

Case report/series	Case	Affected sites	Pre-Tx surgical interval (weeks)	Pre-Tx surgical procedures (n)	Pre-Tx Derkay score	Dose mg/kg	Initial dosing interval (weeks)	Final dosing interval (weeks)	Tx- cycles	Follow-up (months)	Post-Tx surgical interval (months)	Post-Tx surgical procedures (n)	Post-Tx Derkay score	Complications
Nagel et al. 2009	1	Trachea	8	NR	NR	10	3	3	8	NR	NA	0	NR	None
Mohr et al. 2014	1	Larynx, trachea, lung	NR	> 30	NR	10	2	16	16	27	NA	0	NR	HT
	2	Larynx	24	2	NR	10	3	NR	3	NR	NR	1**	NR	None
	3	Larynx	NR	16	NR	10	NR	NR	6	NR	> 12	1	NR	None
	4	Larynx, trachea, lung	NR	> 30	NR	5	2	12	9	NR	17	1	NR	None
	5	Nasopharynx, sinuses	NR	6	NR	15	3	8	6	NR	NA	0	NR	None
Bedoya et al. 2017	1	Trachea, lung	NR	5	NR	5	2	6	> 11	NR	NA	0	NR	HP
	2	Larynx, trachea, lung	NR	Multiple	NR	10	2	3	NR	NR	NA	0	NR	HT
Best et al. 2017	1	Larynx, trachea, lung	3	NR	NR	10	3	8	11	33^c^	3	NR	NR	None
	2	Larynx, trachea, lung	1–4	NR	NR	10	4	8	NR	33^c^	4	NR	NR	PU
	3	Larynx	4–6	NR	NR	10	2	12	NR	33^c^	NA	0	NR	None
	4	Larynx, trachea, lung	4	NR	NR	5	3	3	NR	33^c^	6	NR	NR	None
	5	Larynx, trachea, lung	6	NR	NR	10	3	6	NR	33^c^	NA	0	NR	HP
	6	Larynx, trachea, lung	6	NR	NR	10	3	12	NR	33^c^	3	NR	NR	None
	7	Lung	12	NR	NR	5	3	8	NR	33^c^	NA	0	NR	None
	8	Trachea, lung	48	NR	NR	10	3	6	NR	33^c^	NA	0	NR	None
Zur et al. 2017	1	Larynx, trachea, lung	1–4	± 500	NR	10	4	14	9	15	NR	2	NR	PU, HT
Carnevale et al. 2019	1	Larynx, trachea, lung	NR	Multiple	NR	10	4	10	NR	48	NR	2	NR	PU
	2	Larynx, trachea	NR	47	NR	5^b^	3	3	8	19	NA	0	NR	None
Cuestas et al. 2019	1	Trachea, lung	4	2	NR	10	4	12	6	6	NA	0	NR	None
Baday et al. 2020	1	Larynx, trachea	6.6	75	8	10	4	8	12	NR	NA	0	2	PU
	2	Larynx	8.6	15	5	10	4	8	13	NR	13	1	0	None
	3	NR	8.2	35	5	10	4	8	10	NR	NA	0	1	ET
Gates et al. 2020	1	Larynx, trachea	NR	3	15	10^b^	4	6	18*	24	NR	18*	14	None
Hamdi et al. 2020	1	Larynx, trachea	4	NR	NR	10	3	3	NR	54	7.8	7	NR	None
	2	Larynx, trachea	4	34	NR	10	-	-	1	NR	12	1	NR	None
Tkaczuk et al. 2020	1	Larynx	NR	4^a^	NR	15	3	6	5	7.2	NR	1	NR	ET, HT, creat
	2	Larynx, trachea	NR	2^a^	NR	15	3	8	15	14.2	NR	2	NR	HT, HA
	3	Larynx, trachea, lung	NR	6^a^	NR	10	3	3	3	15.1	NR	3	NR	HT, TP
	4	Larynx, trachea	NR	4^a^	NR	15	3	6	17	15.7	NR	1	NR	HT, HTY
	5	Larynx, trachea	8–12	5^a^	NR	15^b^	3	6	5	16.4	NA	0	NR	None
	6	Larynx, trachea	NR	2^a^	NR	15	3	3	2	4.4	NA	0	NR	DG
	7	Larynx	NR	9^a^	NR	15^b^	3	6	10	8.7	NA	0	NR	None
	8	Larynx	NR	2^a^	NR	15^b^	6	8	6	6.8	NA	0	NR	HT, HA, creat
	9	Larynx	NR	6^a^	NR	15^b^	3	8	7	7.8	NR	1	NR	ET, HA
	10	Larynx	NR	4^a^	NR	15	3	6	13	15.6	NA	0	NR	None
	11	Larynx	NR	4^a^	NR	15	3	8	17	14.3	NA	0	NR	NA
	12	Larynx	NR	2^a^	NR	10	3	6	10	8.8	NA	0	NR	None
	13	Cavum nasi/oris, larynx	NR	3^a^	NR	15^b^	3	6	11	9.5	NA	0	NR	HT
	14	Larynx, trachea, lung	NR	11^a^	NR	15	3	6	6	8.2	NR	1	NR	PM
Enrique et al. 2021	1	Larynx, trachea	8	32	15	10	3	3	3	12	NA	0	0	None
	2	Larynx, trachea	4	5	12	10	3	3	2	NR	NA	0	0	None
	3	Larynx	3	2	7	10	3	3	2	12	NA	0	0	None

*NA* not applicable, *NR* not reported, *PU* proteinuria, *ET* epistaxis, *HP* hemoptysis, *HT* hypertension, *creat* elevated creatinine, *HA* headache, *TP* thrombocytopenia, *HTY* hypothyroidy, *DG* dysgeusia, *NA* nausea, *PM* premature menopause, *Tx* therapy, *n* number

^a^Surgeries 1 year prior to systemic treatment

^b^Dose adapted during treatment

^c^Average number of months

*Surgery combined with post-operative bevacizumab

**Surgery due to malignant transformation

In 72% of the cases (31 out of 43), the duration of follow-up was reported. Calculation resulted in a mean follow-up of 21.6 months (range 4.4–54 months).

Side effects like proteinuria, epistaxis, hemoptysis, hypertension, elevated creatinine level, headache, thrombocytopenia, hyperthyroidism, dysgeusia, nausea, and premature menopause occurred in 44% of the cases (19 out of 43), but were described as mild and self-limiting [9–14, 16, 17, 20, 21]. In the remaining 56% (24 out of 43), no side effects occurred.

### Efficacy of intralesional bevacizumab

The outcomes of the studies describing efficacy of intralesional bevacizumab are presented in Table 4. In 62% of the cases (13 out of 21), the post-bevacizumab surgical interval (range 4–12 weeks) was prolonged when compared to that before (range 2–6 weeks) [15, 18]. One case series did not report the pre- and post-bevacizumab surgical interval, but the initial (range 1.9–17 weeks) and final dosing intervals (range 4.3–21.4 weeks), as well as the pre- (range 3–23) as post-bevacizumab Derkay score (range 0–12) [19].

**Table 4:** Treatment effects of three studies, listed per patient, reporting the use of intralesional bevacizumab as adjunct therapy for recurrent respiratory papillomatosis

Case report/series	Case	Affected sites	Pre-Tx surgical interval (weeks)	Pre-Tx surgical procedures (n)	Pre-Tx Derkay score	Dose, mg	Initial dosing interval (weeks)	Final dosing interval (weeks)	Injections	Follow-up (months)	Post-Tx surgical interval (weeks)	Post-Tx surgical procedures (n)	Post-Tx Derkay score	Complications
Maturo et al. 2010	1	NR	4^a^	12^a^	21	1.25	NR	NR	3	NR	5^a^	NR	6	NR
	2	Larynx	2^a^	22^a^	21	1.25	NR	NR	2	NR	4^a^	NR	23	NR
	3	NR	2.5^a^	NR	13	1.25	NR	NR	1	6	NA	0	NR^d^	NR
Rogers et al. 2013	1–10	NR*	6^a^*	8^a^*	19*	1.25*	2–3*	2–3*	3*	NR	12^b^*	4^b^*	13*	NR
Sidell et al. 2014	1	NR	NR	NR	4	8.6	3.7	7.3	NR^c^	1–1.5	NR	NR	4	None
	2	NR	NR	NR	3	12	6.6	21.4	NR^c^	1–1.5	NR	NR	1	None
	3	NR	NR	NR	23	22.5	2.6	5.3	NR^c^	1–1.5	NR	NR	12	None
	4	NR	NR	NR	13	11.8	1.9	4.3	NR^c^	1–1.5	NR	NR	9	None
	5	NR	NR	NR	14	27.8	6	9.7	NR^c^	1–1.5	NR	NR	7	None
	6	NR	NR	NR	13	11.3	17	NR	NR^c^	1–1.5	NR	NR	0	None
	7	NR	NR	NR	7	8.5	6.4	NR	NR^c^	1–1.5	NR	NR	0	None
	8	NR	NR	NR	9	13.3	NR	NR	NR^c^	1–1.5	NR	NR	3	None

*NA* not applicable, *NR* not reported, *Tx* therapy, *n* number

^a^During the year before bevacizumab injections

^b^After third bevacizumab injection

^c^Maximum of five injections

^d^Patient did not require return to operating room, and so no measurement was taken

*Mean of ten cases

In 43% of the cases (9 out of 21) the duration was reported of follow-up after the initiation of intralesional bevacizumab treatment. Mean follow-up was 1.8 months (range from 1 to 6 months).

The occurrence of side effects was discussed in 38% of the cases (8 out of 21), in which none were found [19].

## Discussion

### Principal findings

The objective of this systematic review was to give an overview of the available literature concerning the efficacy of bevacizumab for RRP, and to differ between the intralesional and systemic treatment strategies. Overall, 95% of the cases showed a considerably prolonged post-bevacizumab surgical interval when treated systemically, and 56% did not require any surgical intervention during follow-up anymore (mean, 21.6 months follow-up). Treatment intralesionally yielded slightly lower efficacy, but in 62% of the cases a prolongation of the post-bevacizumab surgical interval was achieved (mean, 1.8 months follow-up). To interpret these results correctly, several aspects need additional attention: quality of included studies, follow-up, patient selection, outcome parameters, and side effects.

Overall, the individual quality of the included articles was moderate. Twelve studies scored a moderate risk of bias [10–13, 15, 16, 18–23]. We considered these studies to be more reliable compared to the remaining three, which scored a high risk of bias [9, 14, 17]. A higher risk of bias was related to the noncompliance of the following CARE guideline topics: keywords, introduction, timeline, follow-up and outcomes, patient perspective, and informed consent [27].

The follow-up of the included cases was quite short, mean 21.6 months for systemic, and less than 2 months for intralesional application. The ‘natural’ behavior of RRP shows reducing frequencies of surgical interventions over time [28]. This demonstrates that the post-bevacizumab results should be interpreted with caution. However, the objectified prolongation of the post-bevacizumab surgical interval seems to be greater than might be expected from its natural course, which indicates the efficacy of bevacizumab treatment for RRP.

All included patients suffered from a severe type of RRP. Nonetheless, striking results were retrieved after bevacizumab treatment. Therefore, it might be very reasonable to obtain even better results in patients with less severe RRP. Patients treated with intralesional bevacizumab suffered exclusively from JoRRP, which is known to be more aggressive compared to AoRRP [4]. Similarly, it might be reasonable to obtain even better results of intralesional treatment in patients with AoRRP.

According to the 'Systemic Bevacizumab for Treatment of Respiratory Papillomatosis: International Consensus Statement’ [24], two parameters are internationally recognized for objectifying RRP severity: need for tracheotomy and surgery frequency. Thus, the efficacy of bevacizumab can be derived by comparing the surgical interval before and after treatment [24]. Interestingly, one study did not report these surgical intervals [19]. Instead, two other outcome parameters (initial and final bevacizumab dosing interval, and pre- and post-bevacizumab Derkay score) were provided [19]. This detail could influence the overall treatment efficacy as is seen in the group of intralesional bevacizumab. Both parameters showed an improvement after treatment [19]. Consequently, the primarily calculated 62% of cases which showed prolongation of surgical interval after intralesional treatment might be underestimated because of missing data.

Side effects solely occurred in the group of patients treated systemically and were described as mild and self-limiting [9–14, 16, 17, 20, 21]. However, one might discuss whether premature menopause, as reported once [20], is indeed a mild and self-limiting side effect. The lack of serious side effects indicates that the administration of bevacizumab might be safe, but should be seen in the light of lacking long-term follow-ups as well. Thus, based on this systematic review, it is hard to make any conclusions about the safety of bevacizumab treatment for RRP. However, several studies in today’s literature describe manageable side effects of bevacizumab treatment and the overall safety outcomes observed support the tolerability of long-term bevacizumab treatment in a diverse range of tumors [29, 30].

Recently, we published our own experience of bevacizumab treatment for AoRRP [31]. The patient underwent multiple surgical interventions for RRP over several years, but was effectively treated with systemic bevacizumab. The post-bevacizumab surgical interval was considerably prolonged. During 32 months of follow-up, solely one surgical intervention was performed, specifically 24 months after the initiation of treatment. Also, no side effects occurred [31].

Concurrent to our systematic review, another systematic review was published that investigated the efficacy and safety of systemic bevacizumab only for JoRRP, and similar results were found [32]. All patients experienced a considerable improvement of symptoms with a reduced need for surgical intervention during follow up (range 2 months to 5 years). Also, 55% of the patients did not require any surgical intervention after the initiation of bevacizumab anymore. In 30% of the cases, side effects occurred, but were considered as milde or moderate. The remaining 70% did not report any side effects [32]. However, these findings are solely based on cases of JoRRP, and not AoRRP. Furthermore, no intralesional administration of bevacizumab was investigated. As a consequence, only a small sample size of 20 participants was included [32]. To provide a complete overview of the efficacy of bevacizumab for RRP in our study, we included all case reports, case series and retrospective studies available in medical literature concerning systemically and intralesionally administered bevacizumab for JoRRP and AoRRP.

### Strengths and limitations

The main strengths of this study are the systematic approach and its comprehensive multilingual search strategy, which allowed us to identify all relevant articles and available data from the literature. The main limitation is that medical literature mainly describes case reports and case series with small samples size, since bevacizumab is a new therapeutic agent for the treatment of RRP. Another limitation is the fact that true long term results are lacking. This is relevant, as RRP is known to be able to recur after disease free intervals up to decades [28].

## Conclusion and recommendation

The results of this systematic review clearly indicate that systemically and intralesionally administered bevacizumab are effective treatment options for severe RRP. For both, JoRRP and AoRRP, a systemic administration might be the treatment of first choice. Reasons are a higher overall efficacy with a greater prolongation of the post-bevacizumab surgical interval, and the applicability in locations difficult to treat intralesionally or with standard surgical intervention [24]. Further prospective research and clinical trials with long-term follow-up are advocated to elucidate this important topic, and to investigate the safety of this agent.

## Data Availability

Not applicable.

## Appendix 1

Search strategy concerning publications for use of systemic and intralesional bevacizumab in treatment of recurrent respiratory papillomatosis (performed at 21 February 2022).

**Table:**

Database	Terms	Hits
Pubmed	((((((((((((((Laryngeal papilloma*[Title/Abstract]) OR (Larynx papilloma*[Title/Abstract])) OR (Larynxpapilloma*[Title/Abstract])) OR (LP[Title/Abstract])) OR (LRP[Title/Abstract])) OR (Respiratory papilloma*[Title/Abstract])) OR (RRP[Title/Abstract])) OR (Human papillomavirus[Title/Abstract])) OR (HPV[Title/Abstract])) OR (laryngeal neoplasms[MeSH Terms])) OR (Laryngeal papillomatosis[MeSH Terms])) OR (Recurrent respiratory papillomatosis[MeSH Terms])) OR (Papillomavirus Infections[MeSH Terms])) OR (Papillomaviridae[MeSH Terms])) AND (((Avastin[Title/Abstract]) OR (Bevacizumab[Title/Abstract])) OR (Bevacizumab[MeSH Terms]))	122
Embase	('laryngeal papilloma*':ti,ab,kw OR 'larynx papilloma*':ti,ab,kw OR 'larynxpapilloma*':ti,ab,kw OR 'lp':ti,ab,kw OR 'lrp':ti,ab,kw OR 'respiratory papilloma*':ti,ab,kw OR 'rrp':ti,ab,kw OR 'human papillomavirus':ti,ab,kw OR 'hpv':ti,ab,kw OR 'larynx tumor'/exp OR 'larynx papillomatosis'/exp OR 'wart virus'/exp OR 'papillomavirus infection'/exp) AND ('avastin':ti,ab,kw OR 'bevacizumab':ti,ab,kw OR 'bevacizumab'/exp)	595
Cochrane	('laryngeal papilloma*':ti,ab,kw OR 'larynx papilloma*':ti,ab,kw OR 'larynxpapilloma*':ti,ab,kw OR 'lp':ti,ab,kw OR 'lrp':ti,ab,kw OR 'respiratory papilloma*':ti,ab,kw OR 'rrp':ti,ab,kw OR 'human papillomavirus':ti,ab,kw OR 'hpv':ti,ab,kw OR 'laryngeal neoplasms'/exp OR 'papilloma'/exp OR 'papillomaviridae'/exp OR 'papillomavirus infections'/exp) AND ('avastin':ti,ab,kw OR 'bevacizumab':ti,ab,kw OR 'bevacizumab'/exp)	20
